# Effect of a simulation-based workshop on breaking bad news for anesthesiology residents: an intervention study

**DOI:** 10.1186/s12871-017-0374-7

**Published:** 2017-06-14

**Authors:** Vanda Yazbeck Karam, Hanane Barakat, Marie Aouad, Ilene Harris, Yoon Soo Park, Nazih Youssef, John Jack Boulet, Ara Tekian

**Affiliations:** 1 Lebanese American University School of Medicine, P.O. Box: 36, Beirut, Lebanon; 2 Department of Medical Education, University of Illinois at Chicago, Illinois, IL USA; 3 American University of Beirut Medical Center, Beirut, Lebanon; 4 Foundation for Advancement of International Medical Education and Research, Philadelphia, PA USA

**Keywords:** Breaking bad news, Anesthesiology residents, Simulation based education

## Abstract

**Background:**

Breaking bad news (BBN) to patients and their relatives is a complex and stressful task. The ideal structure, training methods and assessment instruments best used to teach and assess BBN for anesthesiology residents remain unclear. The purpose of this study is to evaluate the effectiveness of an education intervention for BBN based on immersive experiences with a high fidelity simulator and role-play with standardized patients (SPs). A secondary purpose is to gather validity evidence to support the use of a GRIEV_ING instrument to assess BBN skills.

**Methods:**

The communication skills for BBN of 16 residents were assessed via videotaped SP encounters at baseline and immediately post-intervention. Residents’ perceptions about their ability and comfort for BBN were collected using pre and post workshop surveys.

**Results:**

Posttest scores were significantly higher than the pretest scores for the GRIEV_ING checklist, as well as on the communication global rating. The GRIEV_ING checklist had acceptable inter-rater and internal-consistency reliabilities. Performance was not related to years of training, or previous BBN experience.

**Conclusion:**

Anesthesiology residents’ communication skills when BBN in relation to a critical incident may be improved with educational interventions based on immersive experiences with a high fidelity simulator and role-play with SPs.

**Electronic supplementary material:**

The online version of this article (doi:10.1186/s12871-017-0374-7) contains supplementary material, which is available to authorized users.

## Background

Breaking bad news (BBN) to patients and their relatives is a complex and stressful task [1, 2]. In anesthesiology, the range of BBN varies from postponing a surgery to intra-operative patient death [3, 4]. The majority of anesthesiologists will experience perioperative death or an adverse event for a patient over the course of their careers [5]. Although these occurrences are rare, they can have substantial psychological and professional consequences for the anesthesiologist, as well as for patients and their families [6]. The difficult decisions in the direct aftermath of an adverse event require involvement of an experienced physician and will be influenced by the emotional impact that an adverse event has on the physician [7]. Having an effective strategy for addressing patients’ or family members’ distress when bad news is disclosed increases the physician’s confidence in this difficult task [8–10].

Formal teaching does not often address communication skills and residents learn BBN “on the job” [11]. This training is highly variable and context specific, as many of the “teachers” themselves have had no formal training [12]. Few studies in the literature describe initiatives to include BBN in the anesthesia curricula [13]. These studies describe the comfort level and the attitudes of residents toward BBN after an educational intervention, but without evaluating the effectiveness of the intervention for development of residents’ skills in BBN. Therefore, it remains unclear what structure, training methods or assessment instruments are best used to teach and assess BBN for anesthesiology residents.

“The GRIEV_ING Death Notification Protocol” is a curriculum developed for death notification in the context of an emergency department setting. The GRIEV_ING “G: Gather, R: Resources, I: Identify, E: Educate, V: Verify, I: Inquire, N: Nuts and Bolts and G: Give” assessment tool is a 27-item instrument that has been used to assess confidence in BBN of emergency medicine residents [14], senior medical students [15], paramedics [16], and pediatric residents [17].

The purpose of this study is to evaluate the effectiveness of an education intervention for BBN for anesthesiology residents modeled on “The GRIEV_ING Death Notification Protocol” and, concurrently, to gather evidence to support the psychometric adequacy of the GRIEV_ING scale scores for assessing the BBN skills of residents.

## Methods

Institutional Review Board approval was obtained from the Lebanese American University and from the University of Illinois at Chicago. Participants were informed that their participation was voluntary; results were anonymized. Written consent was obtained prior to the educational intervention.

### Participants

All PGY3 and PGY4 Lebanese anesthesiology residents were invited to participate in this education intervention. PGY1 and PGY2 residents were not recruited because of the minimal likelihood of BBN encounters at their stage of training. A pre-workshop survey focused on residents’ previous training and experiences with BBN and their self-perceived competence and comfort in BBN, as well as their perspectives about the need for associated training programs (Additional file 1).

### Delivery of the education intervention

The four-hour session was conducted at the Clinical Simulation Center and was organized by two anesthesiologists, a clinical psychologist and a simulation educator. Following an orientation to the simulation setting, participants were randomly divided into groups of four. Each group participated in an immersive experience (case 1, described below) with the high fidelity simulator (HFS) (iStan, METI, CAE). This was followed by an individual encounter with an SP. After all residents participated in case 1, they participated in a teaching intervention. The intervention consisted of short lectures provided by the anesthesiologist and the clinical psychologist, role-play and group discussions. Both educators facilitated the debriefing and encouraged the participants and SPs to reflect on what went well in the conversation and to make suggestions for improvement. Following this teaching intervention, all participants were again randomly divided into groups of four and each group participated in case 2 (described below), followed by an individual encounter with an SP (Table 1).

**Table 1:** Study timeline

1. Pre workshop survey 2. Orientation to all groups 3. Participants by groups of 4 undergo an immersive scenario (case 1), followed by individual interaction with an SP. The SP completed the global rating and the GRIEV_ING checklist. 4. Teaching intervention to all groups 5. Participants by groups of 4 undergo the immersive scenario (case 2), followed by individual interaction with an SP. The SP completed global rating and GRIEV_ING checklist. 6. Post workshop survey 7. Anesthesiologist independently rate the videotaped encounters using the GRIEV_ING checklist assessment form	

All encounters with family members were conducted in Arabic, the national language and videotaped. At the end of the session, participants completed a survey about their perceived ability and comfort in BBN (Additional file 2).

### The scenarios

Two scenarios describing situations of unexpected intraoperative anesthetic complications were developed by experienced clinicians (anesthesiologists), assisted by experts in simulation-based medical education (LAU-CSC educators) and a clinical psychologist. Both scenarios were based on the complication being from a purely anesthetic origin and not from the surgery. Intraoperative death from a purely anesthetic complication is an extremely rare event. The most common encounter for BBN for an anesthesiologist is caused by intraoperative complications that lead to sending the patient to the intensive care unit. Both cases occurred during a routine anesthesia induction in a healthy patient.

In case one, a young patient admitted for a surgery of the knee developed an anaphylactic reaction during the induction of anesthesia. In case 2, a middle-aged patient admitted for back surgery developed a sudden onset of atrial fibrillation after the induction of anesthesia. In both cases, and following the resuscitation efforts of the participants, the patient’s critical state was stabilized, which allowed his transfer to the intensive care unit. Each participant was then asked to inform a patient’s family member about this unexpected complication.

### The assessment instrument

The GRIEV_ING is a 27-item instrument developed to focus on 8 competency areas concerning death notification (Additional file 3). This instrument can be divided into subscales that measure different components of the BBN skill: preparation, delivery and wrap-up [17]. After a thorough review by the organizing team, the general structure of this checklist was maintained, while some descriptors were modified to accommodate the notification of an adverse event or death in the anesthesiology context. This modification of the instrument was followed by piloting a few scenarios of BBN with SPs; the wording was further modified, based on feedback. The checklist was also sent to three anesthesiology program directors to ensure its applicability for assessing BBN skills. In addition to this GRIEV_ING checklist assessment form, participants were also assessed for communication skills by the SPs using a global rating instrument that was adapted from the GRIEV_ING Death Notification Protocol” (Additional file 4).

### The raters

Five SPs were trained by the organizing team. SPs participated in a two-hour training session for their roles as family members and a four-hour training and calibration session to prepare them for their rater role. Immediately preceding the workshop, SPs participated in a review session to ensure that all instructions were clear. The two anesthesiologists independently rated the videotaped encounters using the GRIEV_ING checklist assessment form.

### Study design

Residents’ skills in BBN were assessed with an SP encounter at two separate time points: before the teaching intervention with case 1 (anaphylaxis) and immediately post-intervention with case 2 (cardiac event). Residents were not assessed on the way they managed the intra-operative events, but only on the way they delivered the bad news to the family member. However, they were not informed about this fact to preserve the realism and the total immersion required by our intervention. We chose to use two different cases, one at time 1 and the second at time 2, for a number of reasons. First, given the educational intervention, it would be difficult, logistically, to conduct the 2 different simulations at the same time point. Second, and more important, we did not want our results to be confounded by case familiarity. While the patient presentation and associated management was different at the 2 time points, the resident task, breaking bad news, was the same. As such, the two assessments, before and after the intervention, were considered to be of equivalent difficulty.

Our primary hypothesis was that, compared to baseline, residents would perform significantly better on the skills associated with BBN after the educational intervention. Considering the GRIEV_ING total checklist score, a paired sample t-test (β = .2, α = .05), and a medium effect size of 0.5, our required sample size would be 25.

### The assessments

Standardized patients completed both the modified GRIEV_ING checklist and the communication global rating assessment. The GRIEV_ING checklist is a binary assessment tool with equal credit given for each item performed. The communication global rating was provided on a scale from 1 = poor to 5 = excellent. To measure inter-rater reliability, six encounters from case 1 and from case 2 were randomly sampled and rated by the two anesthesiologists and the psychologist.

### Data analysis

The intraclass correlation coefficient (ICC) was used to evaluate inter-rater reliability of the GRIEV_ING scores for each case, both by dimension and overall. Raters scored each GRIEV_ING assessment item on a yes/no basis (1 = performed item, 0 = did not perform item). A total score for each case was calculated by summing all items. The percentage of performed items was also calculated for all residents. The internal-consistency reliabilities (Cronbach alpha) for the communication global rating and the GRIEV_ING checklist were calculated for both cases. A paired-samples *t*-test was used to compare changes in mean trainee scores before and after the intervention. This was done separately for the total checklist scores and for the global communication rating scale. Pearson’s correlation coefficient was used to quantify the association between the GRIEV_ING checklist scores and the communication global rating scores.

Survey data were summarized by frequency counts and means. Data were analyzed using the Statistical Package for Social Sciences (SPSS) version 21.

## Results

Out of 32 PGY3 and PGY4 Lebanese residents, 16 (50%) participated in our education activity. Nine (9) were PGY-3 (seven females, two males) and seven were PGY- 4 (two females, five males). Three (3) PGY-3 (two females, one male) and three PGY-4 (one female, two males) residents participated in the focus group interview.

Performance in case 1 (pre-test) did not vary in relation to the year of training (14.7 ± 3.8 for PGY3 and 16.6 ± 4.0 for PGY4, *P =* 0.34), to any previous received training (15.9 ± 3.5 and 15.0 ± 4.5, *P =* 0.66), or on any previous BBN encounters (17 ± 3.9 for ≤5 encounters and 14.3 ± 3.7 for ≥5 encounters, *p =* 0.18).

Pretest scores were low on the GRIEV_ING checklist as well as on the communication global rating, with residents scoring consistently below 65%. Mean Percent of Items Performed Correctly for the Griev_ing Checklist and the Communication Global Rating of the Pretest and Posttest Scores.

The mean percent of items performed correctly in the post-test score for the GRIEV_ING checklist was significantly higher than the pre-test score (82.6 ± 13.0 and 53.4 ± 22.2, *P* < .001, effect size 2.1) In addition, the mean percent of items performed correctly in the post-test score for the communication global rating was significantly higher than the pre-test score (84.7 ± 10.5 and 64.5 ± 12.9, *P* < .001, effect size 1.7) (Table 2).

**Table 2:** Descriptive statistics: mean percent of items performed correctly for the Griev_ing checklist and the communication global rating of the pretest and posttest scores

	Pretest (*n* = 16) Mean (SD)	Posttest (*n* = 16) Mean (SD)	Effect size	*P*-value
GRIEV_ING checklist score	55.3 (12.6)	80.7 (11.5)	2.1	< .001
Communication global rating	64.5 (12.9)	84.7 (10.5)	1.7	< .001

For the GRIEV_ING checklist scores, the intraclass correlation coefficients (ICCs) were 0.96 and 0.83 for case 1 and 2 respectively while the internal-consistency reliabilities were 0.67 and 0.65 for case 1 and 2 respectively (Table 3). For the communication global rating, Cronbach’s alpha was 0.70 for case 1 and 0.79 for case 2.

**Table 3:** Intraclass Correlation Coefficient (ICC) and the Internal-Consistency Reliability (Cronbach alpha) for the Griev_ing Checklist for the pretest and posttest scores

	Pretest	Posttest
ICC	0.96	0.83
Cronbach’s alpha	0.67	0.65

There was a positive correlation (Pearson coefficient) between the GRIEV_ING checklist and the communication global rating for both cases (*r* = 0.82, *n* = 16, *P* < 0.001 for case 1 and *r* = 0.34, *n* = 16, *p* = 0.19 for case 2).

Nine of the 16 residents (56.2%) rated their competence in BBN as good and very good before the workshop. This number increased to 15 (93.7%) after the session. Ten of the 16 residents (62.5%) felt comfortable in BBN before the workshop; all of them felt comfortable BBN after the workshop.

## Discussion

Medical education is currently shifting toward outcomes-based assessment of residents and fellows as evidenced by the Next Accreditation System and the Milestone project developed by the Accreditation Council for Graduate Medical Education (ACGME) and by the CanMEDS [18, 19]. Residencies are asked to evaluate trainees on developmentally based educational achievements in the core and subcompetencies over time, including interpersonal and communication skills. However, formal teaching often does not address communication skills. Moreover, exposure to patients in a clinical environment with ad hoc educational sessions is not sufficient to create competent healthcare practitioners in the required competencies, namely for BBN, a skill that is not often performed by residents. Developing simulation-based educational interventions may help residents acquire the skills to deal with difficult communication challenges such as BBN [20].

Our study demonstrated that anesthesiology residents’ communication skills when BBN in relation to an adverse event improved significantly following an education intervention based on immersive experiences with an HFS and role-play with SPs. We also gathered evidence to support the psychometric adequacy of the GRIEV_ING instrument scores for assessing the acquisition of BBN skills. For both cases, the internal-consistency reliability (Cronbach’s alpha) for the GRIEV_ING checklist and for the communication global rating showed acceptable values. In addition, the intraclass correlation coefficient (ICC), used to assess rater reliability, yielded acceptable values for both cases, consistent with guidelines proposed by Landis and Koch [21].

This is one of the few studies examining BBN skills in anesthesiology residents and its results have important implications for anesthesiology residency programs. Overall scores on the pre-test were low, which clearly demonstrated the need for increased education and repeated interventions for BBN as part of the residency curriculum.

Anesthesiology is somewhat unique with respect to the conditions of patient care [22]. Unlike physicians in other specialties, patients do not often choose their anesthetic provider. As a result, patients and families may not have the same comfort and trust when communicating with the anesthesiologist [23]. Death and serious adverse events associated with anesthesia are rare, but when they occur, they usually arise suddenly and unexpectedly. Unlike physicians in some fields of medicine who may get ready in advance for BBN, anesthesiologists cannot avoid such situations, since they have to immediately announce the death or the occurrence of an intra-operative adverse event to the family of an anesthetized patient [11]. BBN in this context lacks the benefits of an ongoing professional relationship with the patient and is done with the patient’s anxious family. This unique situation, in addition to little formal training in BBN, as well as limited exposure for residents in BBN, underscores the need for simulation training to supplement these rare real-life learning experiences.

Our educational intervention, both the classroom and simulation components, was based on a review of the educational literature related to delivering bad news [24, 25]. The most effective interventions were those that provided opportunities for learners to discuss concerns, practice, and receive feedback on their skills. In addition, role-playing or simulation can be a key part [26–29]. Offering more than one opportunity to practice and receive feedback so that trainees can try out new behaviors they may not have demonstrated in their first encounter has been shown to be important [30, 31]. It was encouraging to find that the self-confidence and the perceived ability of our participants increased by the end of the workshop.

Our investigation had a number of limitations. First, our educational intervention only included a relatively small number of volunteers. While there was representation from all Lebanese Anesthesiology residency programs, one could still question the generalizability of the findings. Second, we did not include a control group. A controlled study would be necessary to prove that educational intervention and subsequent performance were causally linked. Third, our residents were only measured twice, over a fairly short time period. As a result, we cannot make any inferences regarding the long-term retention of BBN skills. Finally, our results were gathered in a simulated environment. Various studies in simulation education have demonstrated that performance in a simulated environment may not translate to real-life situations [32]. Follow-up studies assessing resident performance in real-life situations are certainly warranted.

## Conclusion

Addressing patients’ or family members’ distress when BBN can increase anesthesiology residents’ confidence in this challenging task and lead to less stress and burnout [33]. Findings from this study indicate that BBN is a teachable skill. The evaluation of our education intervention demonstrates the value of integrating BBN into the curriculum of the anesthesiology residents.

## Additional files

Additional file 1: Survey completed by participants before the workshop. This pre-workshop survey focuses on residents’ previous training and experiences with BBN and their self-perceived competence and comfort in BBN, as well as their perspectives about the need for associated training programs. (DOCX 13 kb)

Additional file 2: Survey completed by participants at the end of the workshop. This post-workshop survey focuses on residents’ perceived ability and comfort in BBN. (DOCX 32 kb)

Additional file 3: The GRIEV_ING Competence Instrument, modified for anesthesiologist use. The GRIEV_ING is a 27-item instrument developed to focus on 8 competency areas concerning death notification. (DOCX 12 kb)

Additional file 4: Relationship and Communication Instrument used by Standardized Patients. This global rating instrument was adapted from the GRIEV_ING Death Notification Protocol” and is used by the SPs to assess the communication skills of participants. (DOCX 15 kb)

## Abbreviations

BBN: Breaking bad news
HFS: High fidelity simulator
ICC: Intraclass correlation coefficient
SPs: Standardized patients
