# Supplementary material for: Effect of a simulation-based workshop on breaking bad news for anesthesiology residents: an intervention study
Source: BMC Anesthesiol. 2017 Jun 14;17:77. doi: 10.1186/s12871-017-0374-7 (PMC5471713; doi:10.1186/s12871-017-0374-7)
Supplement: Supplementary file 1 — Survey completed by participants before the workshop. This pre-workshop survey focuses on residents’ previous training and experiences with BBN and their self-perceived competence and comfort in BBN, as well as their perspectives about the need for associated training programs. (DOCX 13 kb) [file 12871_2017_374_MOESM1_ESM.docx]

Additional file **1.** Survey completed by participants before the workshop

1- Sex: ☐ Male ☐ Female

2- Level of training: ☐ PGY3 ☐ PGY4

3. Have you ever received any education for BBN? ☐ Yes ☐ No
4. If yes, where did you receive education for BBN? Check one or more answers.

☐ In medical school, while learning about medical ethics

☐ In medical school, while learning clinical skills

☐ During seminars or education programs for residents

☐ On the internet or through mass media

☐ By senior residents or staff

☐ Other: Specify _______________________

5. Have you ever delivered bad news to patients or patients' family? ☐ Yes ☐ No

6. If yes, what was the encounter? How many times?

☐ Postponing or canceling a surgery ☐ 1- 4 ☐ 5-10 ☐ >10

☐ Unexpected postop ICU admission ☐ 1- 4 ☐ 5-10 ☐ >10

☐ Intraoperative death ☐ 1- 4 ☐ 5-10 ☐ >10

☐ Other: ……………………………… ☐ 1- 4 ☐ 5-10 ☐ >10

7. If yes, rate your own comfort in dealing with patients' or family members' emotions (e.g., crying, anger, denial)

☐ Very comfortable ☐Quite comfortable ☐ Not very comfortable ☐ Uncomfortable

8. How do you feel about your own ability to break bad news?

☐ Excellent ☐ Very Good ☐ Good   ☐ Poor

9. Do you believe that BBN is a skill that can be taught?

☐ Strongly believe ☐ Believe ☐ Fairly believe ☐ I don’t believe at all

10. Do you believe that training is needed for adequate skill development in BBN during your residency program?

☐ Strongly believe ☐ Believe ☐ Fairly believe ☐ I don’t believe at all

11. Are you willing to attend a training workshop if you have the opportunity?

☐Yes ☐ Not decided ☐ No
