# Supplementary material for: Effect of a simulation-based workshop on breaking bad news for anesthesiology residents: an intervention study
Source: BMC Anesthesiol. 2017 Jun 14;17:77. doi: 10.1186/s12871-017-0374-7 (PMC5471713; doi:10.1186/s12871-017-0374-7)
Supplement: Supplementary file 3 — The GRIEV_ING Competence Instrument, modified for anesthesiologist use. The GRIEV_ING is a 27-item instrument developed to focus on 8 competency areas concerning death notification. (DOCX 12 kb) [file 12871_2017_374_MOESM3_ESM.docx]

Additional file 3**:** The GRIEV_ING Competence Instrument, modified for anesthesiologist use

**Directions for use**: Please indicate whether the physician completed the stated actions, with **Y = completed (Yes) or N = did not complete (No).**

**The Physician...**

**G—Gather**

*1. Ensured that family members were present prior to the delivery of the critical incident/ death notification.*

**R—Resources**

*2. Inquired and facilitated access to supportive resources for the important family members*

*3. Inquired &facilitated access to supportive resources for the anesthesiologist: facility and human support (senior anesthesiologist, surgeon, OR director, consultant medical physician, etc..)*

**I—Identify**

*4. Clearly stated the name of the patient.*

*5. Clearly introduced herself/himself.*

*6. Clearly stated his/her role in the care of the patient.*

*7. Determined the level of knowledge the family member possessed prior to their arrival in the waiting room.*

*8. Provided an appropriate opening statement (i.e., avoided bluntly stating the critical incident/ death of patient).*

*9. Used preparatory phrases to forecast the news of the critical incident/ death.*

**E—Educate**

*10. Clearly indicated the chronology of events leading up to the critical incident/death of the patient.*

*11. Clearly indicated the cause of the critical incident/ death in an understandable manner.*

*12. Used language appropriate for the family members’ culture and educational level.*

*13. Provided a summary of important information to ensure understanding.*

**V—Verify**

*14. Used the terms “critical state” or “dead” or “died.”*

*15. Avoided using euphemisms (indirect terms).*

*16. Avoided medical terminology/jargon or clearly explained such terms when used.*

*17. Was attentive and not rushed in his/her interaction with the family member/s.*

*18. Paused to allow the family to assimilate the information before discussing details*.

**I—Inquire**

*19. Allowed the family member/s to react to the information and ask questions or express concerns.*

*20. Encouraged the family member/s to summarize important information to check for understanding.*

*21. Immediately but appropriately corrected any misconceptions of the family member/s.*

**N—“Nuts and bolts”**

Explained and addressed the following details of the patient's care adequately.

*22. Transfer to ICU and ICU stay.*

*23. Need for further investigation and treatment.*

*24. Accessibility and schedule visit in ICU.*

**G—Give**

*25. Established personal availability to answer questions for the family member/s at a later date.*

*26. Provided family member/s the appropriate information to contact him/he at a later time.*

*27. Provided the family member/s with appropriate information to contact the care provider team (ICU physician, consulted physician, social worker, …).*

**Total: ---- /27 points**
