# Supplementary material for: Effect of a simulation-based workshop on breaking bad news for anesthesiology residents: an intervention study
Source: BMC Anesthesiol. 2017 Jun 14;17:77. doi: 10.1186/s12871-017-0374-7 (PMC5471713; doi:10.1186/s12871-017-0374-7)
Supplement: Supplementary file 4 — Relationship and Communication Instrument used by Standardized Patients. This global rating instrument was adapted from the GRIEV_ING Death Notification Protocol” and is used by the SPs to assess the communication skills of participants. (DOCX 15 kb) [file 12871_2017_374_MOESM4_ESM.docx]

Additional file 4: Relationship and Communication Instrument used by Standardized Patients.

HOW WAS THE RESIDENT YOU JUST SAW AT:

1 2 3 4 5 Greeting you warmly; never crabby or rude.

1 2 3 4 5 Treating you with modesty and professionalism

1 2 3 4 5 Showing interest in you as a person.

1 2 3 4 5 Listening carefully; asking thoughtful questions; not interrupting while you are talking.

1 2 3 4 5 Encouraging you to ask questions; answering them clearly; never avoiding your questions.

1 2 3 4 5 Using easily understood words when explaining the situation; explaining any technical medical terms in plain language.

WITH THIS RESIDENT, TO WHAT DEGREE DID YOU FEEL:

1 2 3 4 5 Respected

1 2 3 4 5 Comfortable/At ease

1 2 3 4 5 Understood

**Total: ---- /45 points**

***SCALE: 1=poor, 2=fair, 3=good, 4= very good, 5= excellent***
