# Supplementary material for: Effect of a simulation-based workshop on breaking bad news for anesthesiology residents: an intervention study
Source: BMC Anesthesiol. 2017 Jun 14;17:77. doi: 10.1186/s12871-017-0374-7 (PMC5471713; doi:10.1186/s12871-017-0374-7)
Supplement: Supplementary file 4 [file 12871_2017_374_MOESM2_ESM.docx]

Additional file 2. Survey completed by participants at the end of the workshop

1. After attending this workshop, rate your own comfort in your future encounters or family members' emotions (e.g., crying, anger, denial)?

☐ Very comfortable ☐Quite comfortable ☐ Not very comfortable ☐ Uncom

2. After attending this workshop, how do you feel about your own ability to break your future encounters with patients or family members?

☐ Excellent ☐ Very Good ☐Good ☐ Poor

3- Did the workshop help you reduce the stress levels when you will deliver bad n patient/family?

☐ Very much ☐ Acceptable level ☐ Not so much ☐ Not

5- Did the workshop change your perceptions regarding delivering bad news?

☐ Very much ☐ Acceptable level ☐ Not so much ☐ Not

6- What were the most important things you learned during this workshop?

7- What were the major strengths of this workshop?

8- What are the major improvements that could be made in this workshop?
